# Supplementary figures and images for: Size-age population structure of an endangered and anthropogenically introgressed northern Adriatic population of marble trout (Salmo marmoratus Cuv.): insights for its conservation and sustainable exploitation
Source: PeerJ. 2023 Mar 17;11:e14991. doi: 10.7717/peerj.14991 (PMC10026717; doi:10.7717/peerj.14991)

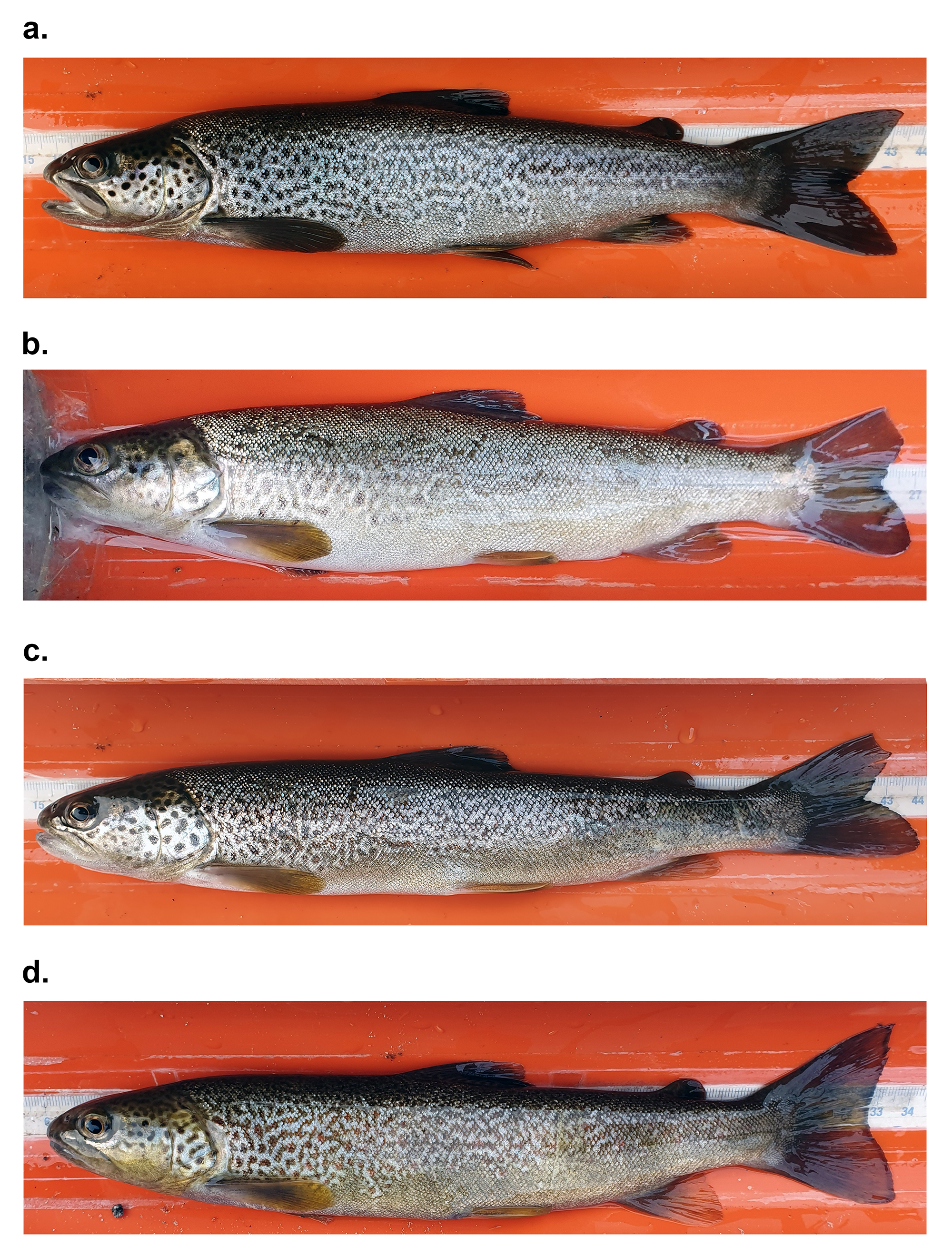

Supplement: Supplemental Information 1 — a, male, 27.2 cm TL, 147 g, Toce River, Oira (46°10′23.4″N 8°19′10.2″E; TOR3; Fig. 1), age: 34 months (mo). b, female, 26.8 cm TL, 191 g, Toce River, Oira (46°10′22.6″N 8°19′08.4″E; TOR3), 40 mo. c, female, 28.3 cm TL, 156 g, Oira (as in a.; TOR3), 34 mo. d, female, Toce River, Oira (as in a.; TOR3), 27.4 cm TL, 149 g, 34 mo. [file peerj-11-14991-s001.jpg]

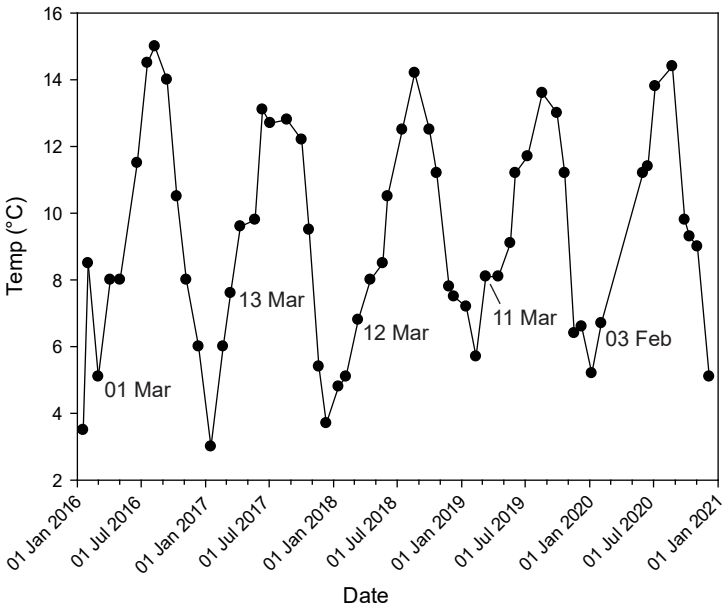

Supplement: Supplemental Information 2 — Site ~5.5 km upstream of the confluence with the Strona Torrent (45°58′29.1″N, 8°25′18.5″E, TOR2; Fig. 1). Temperature sampling dates closest to the 1st of March are indicated on the plot, to mark the annual temperature increase, following the deposition of the winter band (annulus) in the marble-trout scales. The river’s monthly average water temperature (monthly measurements) is ~9.5 °C (2001–2019; Candoglia weather station, Laboratorio di Idrochimica, CNR-IRSA Verbania, CIPAIS; Michela Rogora, pers. comm.). [file peerj-11-14991-s002.pdf]

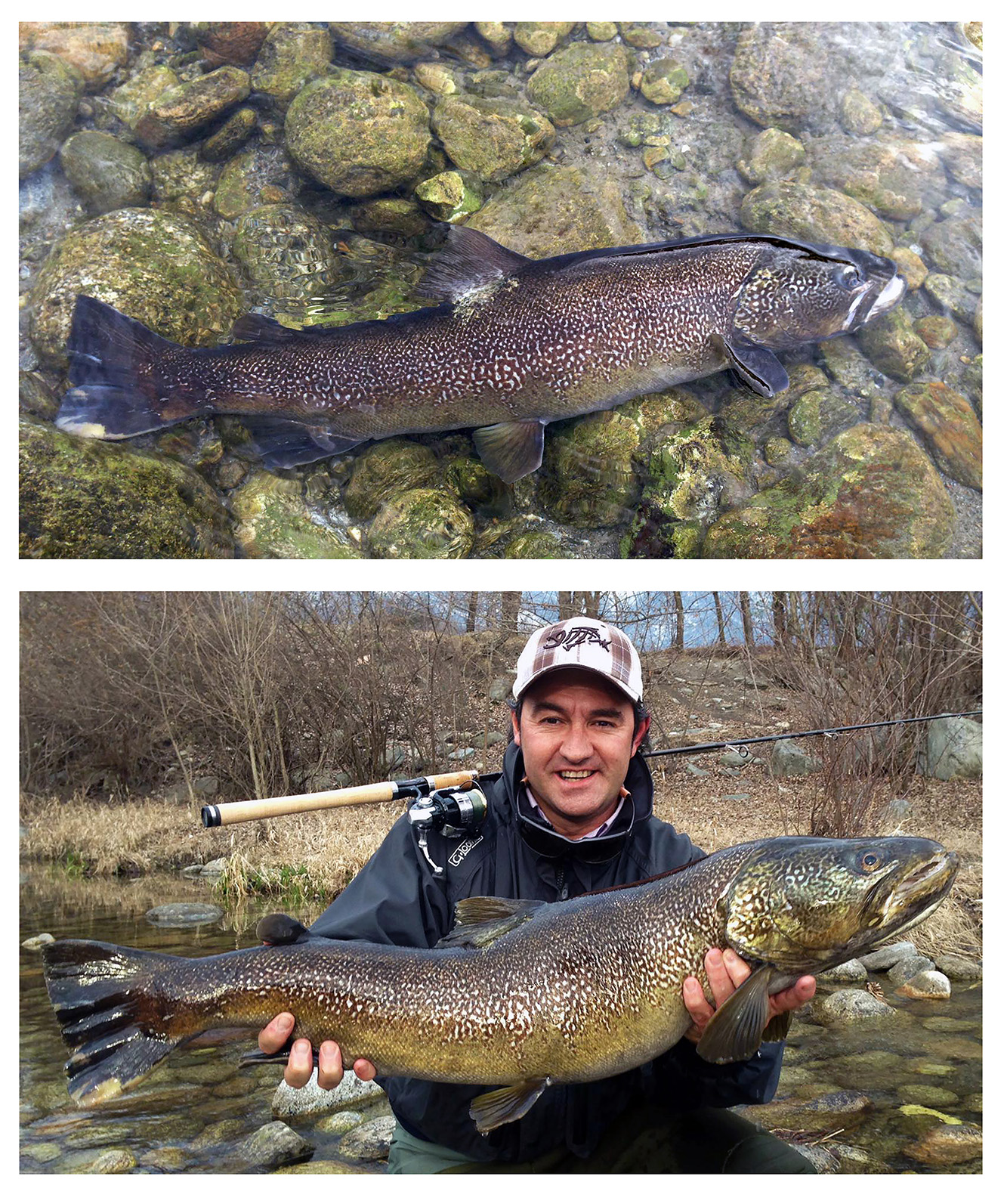

Supplement: Supplemental Information 3 — The fish was caught near Lake Tana, downstream of the confluence between the Toce River and the Diveria Torrent (TOR3; Fig. 1); courtesy of Massimiliano Ghibaudo. [file peerj-11-14991-s003.jpg]

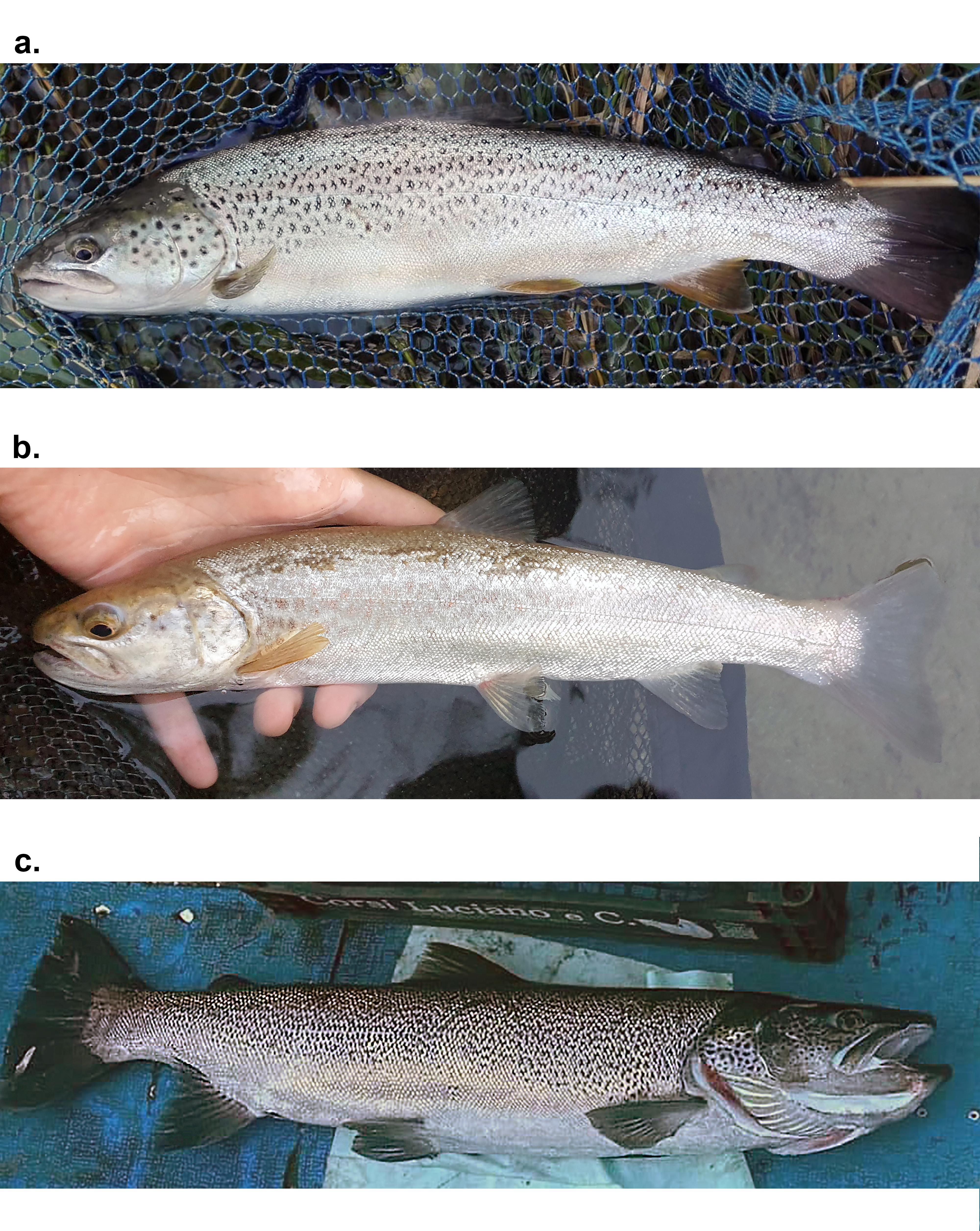

Supplement: Supplemental Information 4 — a. 45.0 cm TL, 1,130 g, 4.5 years of age (m, s), Toce River, Prata (46°01′23.6″N 8°16′55.3″E; TOR2; Fig. 1); b. 29.5 cm TL, 250 g, 2.4 years (m, s, st, l), Toce River, Beura (46°05′22.7″N 8°18′00.5″E; TOR2); c. 85.0 cm TL, 4,600 g, 9.8 years (m, st, l), Lake Maggiore, off Feriolo town (approximately 45°55′43″N 8°29′12″E). [file peerj-11-14991-s004.jpg]

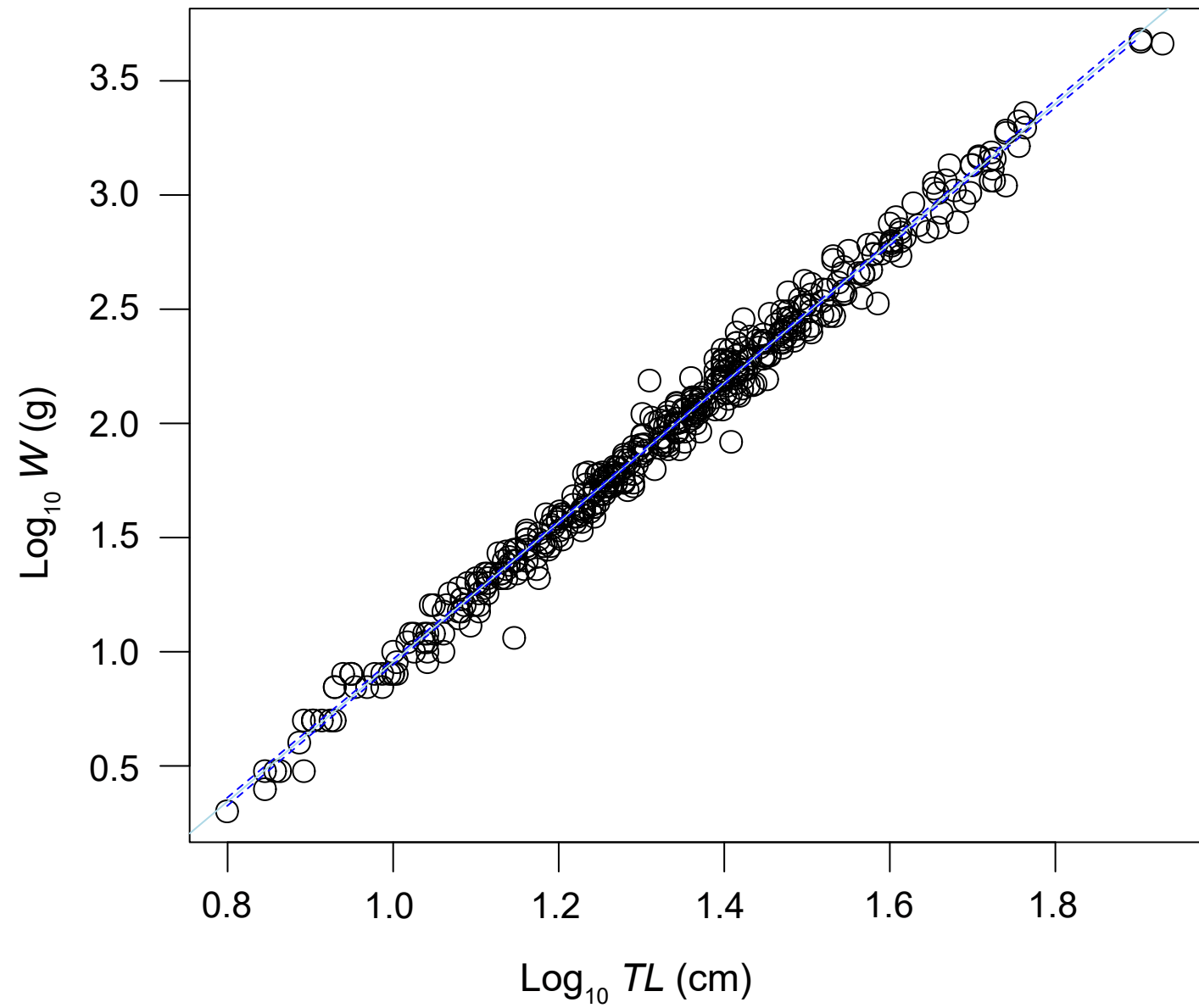

Supplement: Supplemental Information 5 — Linear regression (grey line) of the logarithms of body mass (response variable, W) on the logarithms of length (explanatory variable, TL); dashed lines: 95% CI; adjusted r2 = 0.988, p < 0.0001 (n = 451). [file peerj-11-14991-s005.pdf]

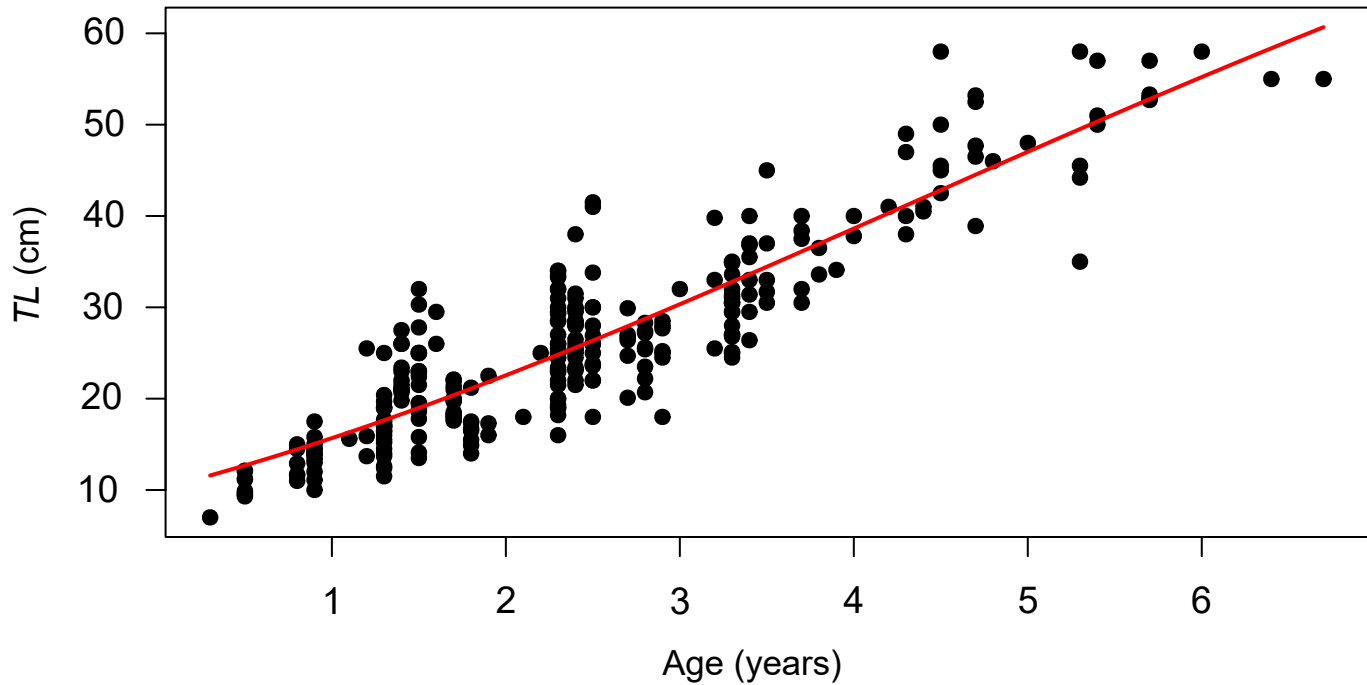

Supplement: Supplemental Information 7 — Gompertz age-specific growth-model without the three largest individuals (n = 292 individuals). [file peerj-11-14991-s007.pdf]

**a**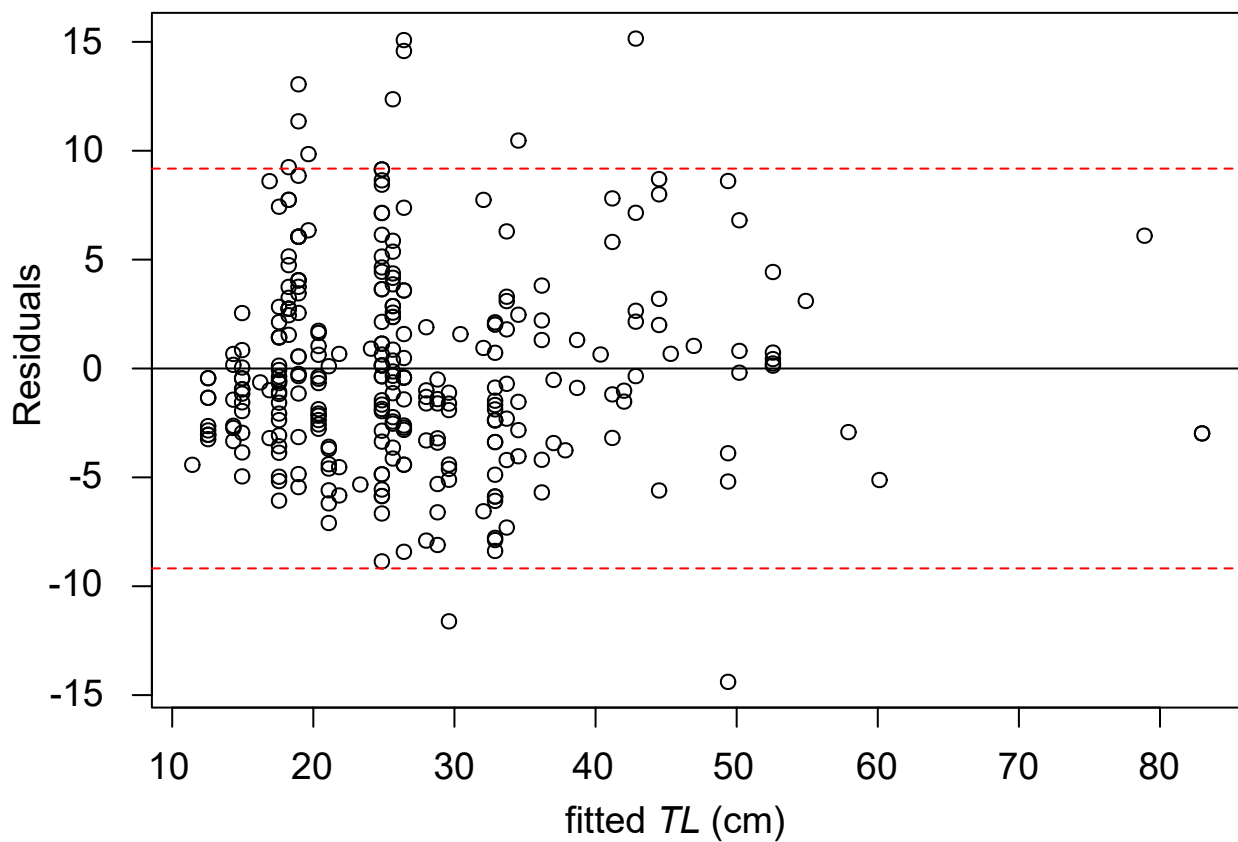**b**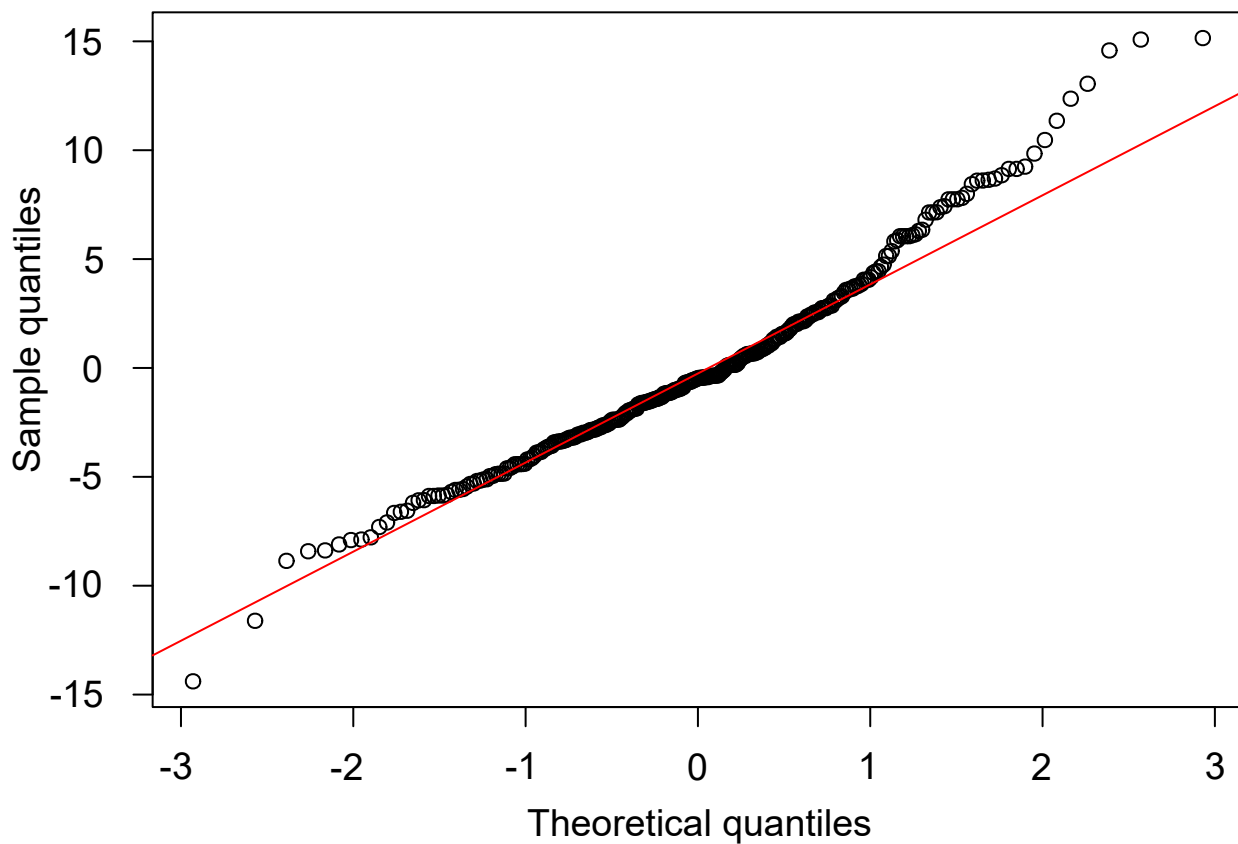

Supplement: Supplemental Information 8 — a. Residual plot of the selected Gompertz model; hatched red lines: 2s.d. interval around mean. b. Normal Q-Q plot of the selected Gompertz model, testing the normal distribution of the residuals. [file peerj-11-14991-s008.pdf]
