# Supplementary material for: Size-age population structure of an endangered and anthropogenically introgressed northern Adriatic population of marble trout (Salmo marmoratus Cuv.): insights for its conservation and sustainable exploitation
Source: PeerJ. 2023 Mar 17;11:e14991. doi: 10.7717/peerj.14991 (PMC10026717; doi:10.7717/peerj.14991)
Supplement: Supplemental Information 6 — Growth trajectories and residual plots (n = 295 individuals). [file peerj-11-14991-s006.pdf]

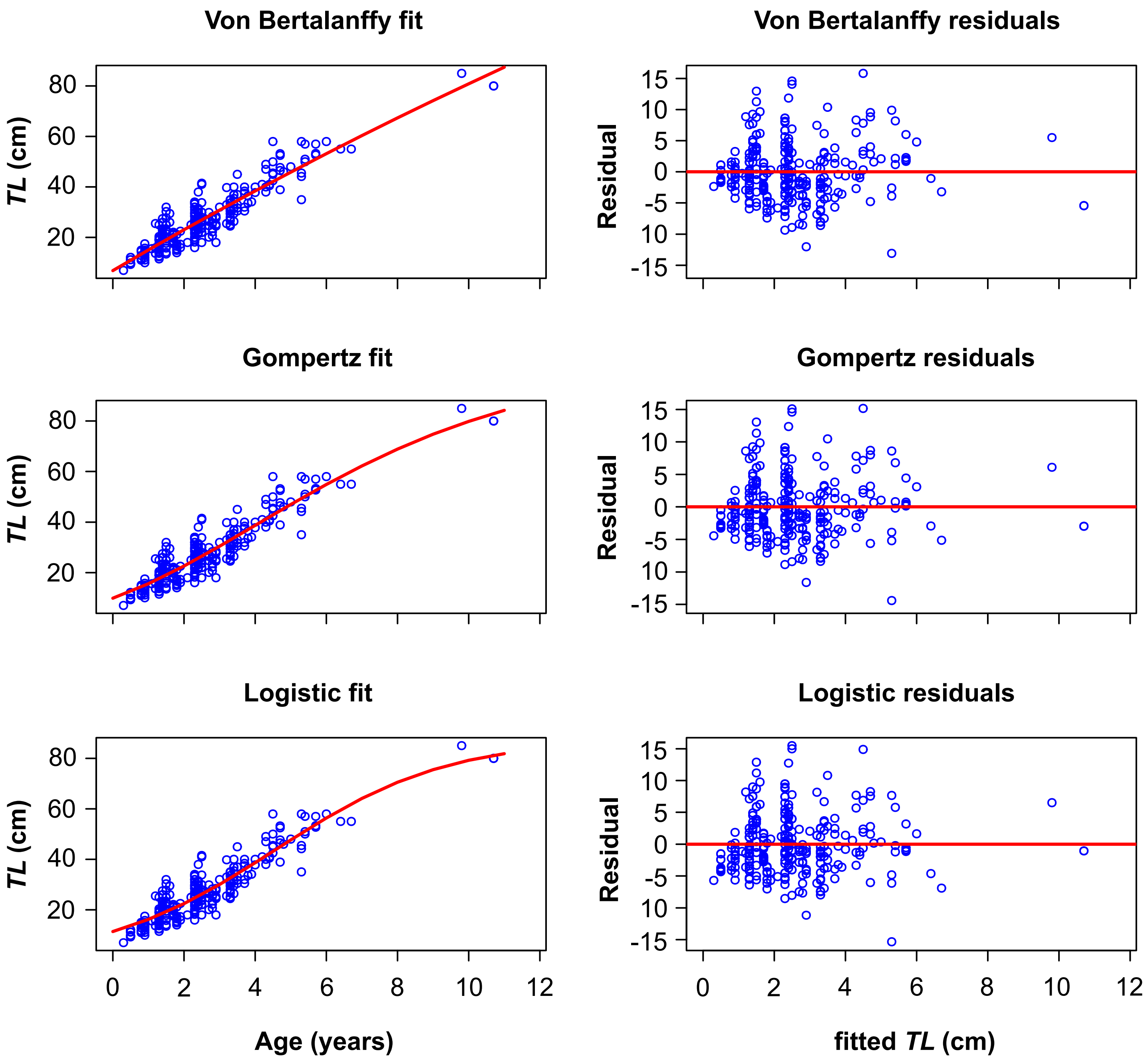

Von Bertalanffy fit
Von Bertalanffy residuals
Gompertz fit
Gompertz residuals
Logistic fit
Logistic residuals
TL (cm)
Residual
80
60
40
20
15
10
5
0
-5
10
-15
0
2
4
6
8
10
12
Age (years)
fitted TL (cm)
