# Supplementary material for: Size-age population structure of an endangered and anthropogenically introgressed northern Adriatic population of marble trout (Salmo marmoratus Cuv.): insights for its conservation and sustainable exploitation
Source: PeerJ. 2023 Mar 17;11:e14991. doi: 10.7717/peerj.14991 (PMC10026717; doi:10.7717/peerj.14991)
Supplement: Supplemental Information 9 — Scale reading and age determinations. Climate and temperature regimes of the Toce River and its effects on aging in S. marmoratus. [file peerj-11-14991-s009.docx]

**Supplementary Note S1. Scale reading and age determinations. Climate and temperature regimes of the Toce River and its effects on aging in *S. marmoratus***

The Toce River catchment’s climate is temperate (latitudinal range: 45°55’ N‒46°28’ N) and is characterized by an average rainfall of ~1,400 mm yr^-1^ (ADBPO, 2018), with two maxima during spring and autumn, and two minima during summer and winter (Saidi et al., 2014). The age of *S. marmoratus* individuals was estimated from scale reading, knowing four lines of evidence. First, breeding season in the Toce River (half-October to half-November; M.I., pers. obs.). Second, number of degree-days (DD) needed for hatching (425‒441 DD, Loro & Zanetti, 1991; 450 DD, Turin, 2000; 400 DD, Zerunian, 2004; Kottelat & Freyhof, 2007; 420‒500 DD in local VCO hatcheries, G.R.A.I.A., 1999). Third, approximate number of degree-days (DD) needed for the first development of scales in the dorsolateral region of the body, after the fry leave the redd and start feeding (140‒180 DD from spawning, Kottelat & Freyhof, 2007). Evidence from other salmonids shows that additional time after the fry start feeding is typically needed for first-scale development. In cold waters, this could occur after up to 2 months [e.g., South-Alaskan populations of *Oncorhynchus clarkii* (Robertson), Ericksen, 1999]. Considering the annual temperatures of the Toce River (Supplementary Fig. S2), we then added one month (210 DD) to these figures. Fourth, monthly surface-water temperatures at the lower tract of the Toce River in 2016‒2020, upstream of the confluence with the Strona Torrent (*TOR1*, Fig. 1; Michela Rogora, pers. comm.), during half-October to half-March (average ± standard deviation~ 6.9±2.3°C), consistent with (i) an incubation of ~2 months at 420‒500 DD from the start and after the end of the breeding season (half-October to half-January); (ii) ~1 month after hatching at 108‒180 DD for yolk-sac resorption (half-January to half-February); and (iii) ~1 month after yolk-sac resorption at 210 DD for 1^st^-scale development (half-February to half-March) (Supplementary Fig. S2). These calculations would imply a period for 1^st^-scale formation after fertilization of ~120 days (~4 months). Considering a reproductive period of 1 month (half-October to half-November), this would mean from half-February to half-March (median value: 1^st^ of March). The first annulus (winter band: a band of more closely packed circuli) is then assumed to complete its deposition on the 1^st^ of March of the next year, before the annual increase in temperature from 4‒6°C to 6‒7°C (Supplementary Fig. S2), as the young-of-the-year would have still not experienced the stunted growth preceding March. Subsequent annuli would also deposit during the period of stunted growth, before the 1^st^ of March. Therefore, age was determined from the counts of the annuli, knowing the month that the scale was sampled, and counting the number of months that have passed since the deposition of the last annulus (Elliot & Chambers, 1996); age in months was then transformed in decimal years for subsequent analyses.
