# Supplementary material for: Size-age population structure of an endangered and anthropogenically introgressed northern Adriatic population of marble trout (Salmo marmoratus Cuv.): insights for its conservation and sustainable exploitation
Source: PeerJ. 2023 Mar 17;11:e14991. doi: 10.7717/peerj.14991 (PMC10026717; doi:10.7717/peerj.14991)
Supplement: Supplemental Information 11 — n: number of field surveys; %: percentage number of field surveys (n = 64); S: type of sample (A: electrofishing, B: angling); TRV: total water volume sampled (m3) during electrofishing surveys in different intervals of space and time; TOR1‒TOR3: sampled areas in the lower, middle, and upper Toce River basin; Subtot A,B: subtotals for sample A,B; Total: survey data for the whole dataset. [file peerj-11-14991-s011.docx]

**Supplementary Table S2**. Spatiotemporal distribution of field surveys (Table S1); *n*: number of field surveys; *%*: percentage number of field surveys (n= 64); *S*: type of sample (A: electrofishing, B: angling); *TRV*: total water volume sampled (m^3^) during electrofishing surveys in different intervals of space and time; *TOR1‒TOR3*: sampled areas in the lower, middle, and upper Toce River basin; *Subtot A,B*: subtotals for sample A,B; *Total*: survey data for the whole dataset.

|  |  | **2016** | | |  | **2017** | | |  | **2018** | | |  | | **2019** | |  | **2020** | | |  | **Total** | | |
| --- | --- | --- | --- | --- | --- | --- | --- | --- | --- | --- | --- | --- | --- | --- | --- | --- | --- | --- | --- | --- | --- | --- | --- | --- |
|  | ***S*** | ***n*** | ***%*** | ***TRV*** |  | ***n*** | ***%*** | ***TRV*** |  | ***n*** | ***%*** | ***TRV*** |  | ***n*** | ***%*** | ***TRV*** |  | ***n*** | ***%*** | ***TRV*** |  | **n** | **%** | ***TRV*** |
| TOR1 | A | 0 | 0.0 | 0 |  | 4 | 6.3 | 6,450 |  | 0 | 0.0 | 0 |  | 0 | 0.0 | 0 |  | 2 | 3.1 | 3,612 |  | 6 | 9.4 | 10,062 |
| TOR2 | A | 0 | 0.0 | 0 |  | 8 | 12.5 | 2,682 |  | 0 | 0.0 | 0 |  | 0 | 0.0 | 0 |  | 5 | 7.8 | 938 |  | 13 | 20.3 | 3,620 |
| TOR3 | A | 0 | 0.0 | 0 |  | 3 | 4.7 | 660 |  | 0 | 0.0 | 0 |  | 3 | 4.7 | 1,154 |  | 11 | 17.2 | 5,450 |  | 17 | 26.6 | 7,264 |
| Subtot A | | 0 | 0.0 | 0 |  | 15 | 23.4 | 9,792 |  | 0 | 0.0 | 0 |  | 3 | 4.7 | 1,154 |  | 18 | 28.1 | 10,000 |  | 36 | 56.3 | 20,946 |
| TOR1 | B | 0 | 0.0 | 0 |  | 0 | 0.0 | 0 |  | 0 | 0.0 | 0 |  | 0 | 0.0 | 0 |  | 5 | 7.8 | 0 |  | 5 | 7.8 | 0 |
| TOR2 | B | 1 | 1.6 | 0 |  | 0 | 0.0 | 0 |  | 3 | 4.7 | 0 |  | 0 | 0.0 | 0 |  | 10 | 15.6 | 0 |  | 14 | 21.9 | 0 |
| TOR3 | B | 1 | 1.6 | 0 |  | 1 | 1.6 | 0 |  | 2 | 3.1 | 0 |  | 1 | 1.6 | 0 |  | 4 | 6.3 | 0 |  | 9 | 14.1 | 0 |
| Subtot B | | 2 | 3.1 | 0 |  | 1 | 1.6 | 0 |  | 5 | 7.8 | 0 |  | 1 | 1.6 | 0 |  | 19 | 29.7 | 0 |  | 28 | 43.8 | 0 |
| Total | | 2 | 3.1 | 0 |  | 16 | 25.0 | 9,792 |  | 5 | 7.8 | 0 |  | 4 | 6.3 | 1,154 |  | 37 | 57.8 | 10,000 |  | 64 | 100.0 | 20,946 |
