# Supplementary material for: Size-age population structure of an endangered and anthropogenically introgressed northern Adriatic population of marble trout (Salmo marmoratus Cuv.): insights for its conservation and sustainable exploitation
Source: PeerJ. 2023 Mar 17;11:e14991. doi: 10.7717/peerj.14991 (PMC10026717; doi:10.7717/peerj.14991)
Supplement: Supplemental Information 12 — K, t0 = Von Bertalanffy growth coefficient and theoretical age when TL = 0, respectively; G, G′, t0 = Gompertz, logistic growth-rate coefficients, and time at the curves’ inflection points, respectively. [file peerj-11-14991-s012.docx]

**Supplementary Table S3.** Estimated parameters (±1 s.e.) for the tested models. *K*, *t_0_*= Von Bertalanffy growth coefficient and theoretical age when *TL*= 0, respectively; G, G’, *t_0_*= Gompertz, logistic growth-rate coefficients, and time at the curves’ inflection points, respectively.

|  | *TL_inf_* | *K/G/G’* | *t_0_* |
| --- | --- | --- | --- |
| Von Bertalanffy | 381.032±234.82 | 0.02±0.02 | ‒0.83±0.15 |
| Gompertz | 104.97±7.51 | 0.22±0.02 | 3.99±0.36 |
| Logistic | 87.37±3.94 | 0.42±0.02 | 4.56±0.24 |
