# Supplementary material for: Size-age population structure of an endangered and anthropogenically introgressed northern Adriatic population of marble trout (Salmo marmoratus Cuv.): insights for its conservation and sustainable exploitation
Source: PeerJ. 2023 Mar 17;11:e14991. doi: 10.7717/peerj.14991 (PMC10026717; doi:10.7717/peerj.14991)
Supplement: Supplemental Information 13 — n = number of estimated parameters; AICc = AIC value corrected for size sample; Delta AICc = difference between ranked models (a difference of ~2 indicates a clear choice); AICc Wt = AICc weight, or relative likelihood of the model; Cum. Wt = cumulative model weights; LL = model’s log-likelihood. [file peerj-11-14991-s013.docx]

**Supplementary Table S4.** Model selection based on AICc. *n*= number of estimated parameters; *AICc*= AIC value corrected for size sample; *Delta AICc*= difference between ranked models (a difference of ~2 indicates a clear choice); *AICc Wt*= AICc weight, or relative likelihood of the model; *Cum. Wt*= cumulative model weights; *LL*= model’s log-likelihood.

|  | *n* | *AICc* | *Delta AICc* | *AICc Wt* | *Cum. Wt* | *LL* |
| --- | --- | --- | --- | --- | --- | --- |
| Gompertz | 4 | 1742.06 | 0.00 | 0.64 | 0.64 | ‒866.96 |
| Logistic | 4 | 1743.48 | 1.42 | 0.31 | 0.95 | ‒867.67 |
| Von Bertalanffy | 4 | 1747.29 | 5.23 | 0.05 | 1.00 | ‒869.58 |
