# Supplementary material for: Size-age population structure of an endangered and anthropogenically introgressed northern Adriatic population of marble trout (Salmo marmoratus Cuv.): insights for its conservation and sustainable exploitation
Source: PeerJ. 2023 Mar 17;11:e14991. doi: 10.7717/peerj.14991 (PMC10026717; doi:10.7717/peerj.14991)
Supplement: Supplemental Information 14 — Summary of the Gompertz model without the 3 largest individuals (80–85 cm TL). s.e. = standard error; Pr(>|t|) = p-value associated with the t value. [file peerj-11-14991-s014.docx]

**Supplementary Table S5.** Sensitivity test: summary of the Gompertz model without the 3 largest individuals (80‒85 cm *TL*). s.e.= standard error; Pr(>|t|)= p-value associated with the t value.

|  | *Estimate* | *s.e.* | *t value* | *Pr(>\|t\|)* |
| --- | --- | --- | --- | --- |
| *TL_inf_* | 111.948 | 23.227 | 4.820 | 2.3·10^‒06^ |
| G | 0.205 | 0.035 | 5.853 | 1.3·10^‒08^ |
| t_0_ | 4.304 | 1.029 | 4.184 | 3.8·10^‒05^ |
