# Supplementary material for: Size-age population structure of an endangered and anthropogenically introgressed northern Adriatic population of marble trout (Salmo marmoratus Cuv.): insights for its conservation and sustainable exploitation
Source: PeerJ. 2023 Mar 17;11:e14991. doi: 10.7717/peerj.14991 (PMC10026717; doi:10.7717/peerj.14991)
Supplement: Supplemental Information 15 [file peerj-11-14991-s015.docx]

**Supplementary literature**

ADBPO ‒ Autorità di Bacino Distrettuale del Fiume Po. 2018. Linee generali di assetto idraulico e idrogeologico/3.2 - Elaborato Lombardia, Linee generali di assetto idraulico e idrogeologico nel bacino del Toce. Available at http://www.adbpo.it/PAI (accessed 4 July 2022).

Elliot JM, Chambers S. 1996. A guide to the interpretation of sea trout scales. Windermere Laboratory, R&D Report 22. Institute of Freshwater Ecology.

Ericksen RP. 1999. Scale aging manual for coastal cutthroat trout from Southeast Alaska. Spec. Publ. 99-4. Alaska Department of Fish and Game, Division of Sport Fish.

Kottelat M, Freyhof J. 2007. *Handbook of European freshwater fishes*. Cornol: Kottelat M, Berlin: Freyhof J.

Loro R, Zanetti M. 1991. Prove di riproduzione della trota marmorata in provincia di Belluno. *Il Pesce* 4, 25‒29.

Saidi H, Ciampittiello M, Dresti C, Ghiglieri G. 2014. Assessment of trends in extreme precipitation events: a case study in Piedmont (North-West Italy). *Water Resource Management* 29, 63‒80. DOI: 10.1007/s11269-014-0826-5.

Turin P. 2000. The biology and management of marble trout in the province of Padua (North-Eastern Italy). *Quaderni ETP* 29, 67‒70.

Zerunian S. 2004. Pesci delle acque interne italiane. *Quaderni di Conservazione della Natura*, 20. Ministero Ambiente Tutela Territorio – Istituto Nazionale Fauna Selvatica.
